# Using Automatic Speech Recognition to Optimize Hearing-Aid Time Constants

**DOI:** 10.3389/fnins.2022.779062

**Published:** 2022-03-17

**Authors:** Lionel Fontan, Libio Gonçalves Braz, Julien Pinquier, Michael A. Stone, Christian Füllgrabe

**Affiliations:** ^1^Archean LABS, Montauban, France; ^2^IRIT, CNRS, Université Paul Sabatier, Toulouse, France; ^3^Manchester Centre for Audiology and Deafness, School of Health Sciences, University of Manchester, Manchester, United Kingdom; ^4^School of Sport, Exercise and Health Sciences, Loughborough University, Loughborough, United Kingdom

**Keywords:** hearing aids (HAs), age-related hearing loss (ARHL), random search (RS), automatic speech recognition (ASR), compression speed, attack time, release time, random search, automatic speech recognition, hearing aids, age-related hearing loss, compression speed, attack time, release time

## Abstract

Automatic speech recognition (ASR), when combined with hearing-aid (HA) and hearing-loss (HL) simulations, can predict aided speech-identification performances of persons with age-related hearing loss. ASR can thus be used to evaluate different HA configurations, such as combinations of insertion-gain functions and compression thresholds, in order to optimize HA fitting for a given person. The present study investigated whether, after fixing compression thresholds and insertion gains, a random-search algorithm could be used to optimize time constants (i.e., attack and release times) for 12 audiometric profiles. The insertion gains were either those recommended by the CAM2 prescription rule or those optimized using ASR, while compression thresholds were always optimized using ASR. For each audiometric profile, the random-search algorithm was used to vary time constants with the aim to maximize ASR performance. A HA simulator and a HL simulator simulator were used, respectively, to amplify and to degrade speech stimuli according to the input audiogram. The resulting speech signals were fed to an ASR system for recognition. For each audiogram, 1,000 iterations of the random-search algorithm were used to find the time-constant configuration yielding the highest ASR score. To assess the reproducibility of the results, the random search algorithm was run twice. Optimizing the time constants significantly improved the ASR scores when CAM2 insertion gains were used, but not when using ASR-based gains. Repeating the random search yielded similar ASR scores, but different time-constant configurations.

## Introduction

Recent studies have shown that automatic speech recognition (ASR) can be used, in combination with signal-processing algorithms mimicking the effects of hearing loss (HL), to predict the speech-identification performances of older hearing-impaired (OHI) listeners [see Schädler et al. (2015) and Fontan et al. (2020a) for a discussion of the advantages of ASR-based metrics by comparison to other objective measures of speech intelligibility]. This was demonstrated for unaided (Kollmeier et al., 2016; Schädler et al., 2018; Fontan et al., 2020a) and aided (using simulated or real hearing aids; Fontan et al., 2020b; Schädler et al., 2020) speech perception.

Based on these findings, it has been speculated that ASR-based prediction systems could also be used to assess speech-intelligibility benefits resulting from various hearing-aid (HA) configurations. Recently, Fontan et al. (2020c) used an ASR system to evaluate and to improve the insertion gains recommended by the CAM2 HA fitting rule (Moore et al., 2010b). For each of their hearing-impaired (HI) participants, 625 gain functions (corresponding to systematic variations of CAM2 gains by 0, ± 3, or ± 6 dB) were assessed. Each gain function was applied to speech stimuli using an HA simulator. The amplified speech material was then degraded using the HL simulator developed by Nejime and Moore (1997). Based on each participant’s audiogram, both the elevation of hearing thresholds and loudness recruitment were mimicked. Spectral smearing, which is also implemented in the original HL simulator to mimic the loss of frequency selectivity, was not used used by Fontan et al. (2020c), since its simulation resulted in weaker correlations between ASR scores and human speech intelligibility (Fontan et al., 2020a). Finally, the amplified and degraded stimuli were fed to the ASR system for computing recognition scores. Fontan et al. (2020c) compared the benefits associated with the insertion-gain function yielding the highest ASR scores (the “optimized” gains yielding a mean improvement of 13 percentage points) to those obtained with CAM2 gains in a group of OHI participants. Significantly higher human speech-identification scores were observed for speech amplified with optimized gains than for speech amplified according to the gains recommended by CAM2. These significant improvements were observed both for word and sentence materials.

Gonçalves Braz et al. (2022) extended this work and combined ASR with several random-search (RS) algorithms to optimize not only insertion gains but also compression thresholds. This approach is referred to as OPRA-RS, which stands for “Objective Prescription Rule based on ASR and Random Search.” Using slow time constants for the compressor of the simulated HA, optimized insertion gains and compression thresholds were determined for 12 audiometric profiles corresponding to different levels of HL severity. ASR scores yielded by the optimized parameters were significantly higher than those obtained with CAM2 (mean improvements ranged from 2 to 10 percentage points for the different RS algorithms). Significant differences were observed between RS algorithms in terms of ASR score and convergence speed.

A limitation of Gonçalves Braz et al.’s (2022) study is that only one set of time constants was used. However, aided speech intelligibility depends on the attack and release times of the HA compressor (Moore et al., 2011; Hopkins et al., 2012). Small time constants (i.e., “fast” compression) help perceiving rapid changes in loudness, such as those occurring when a weak speech sound (e.g., a consonant) precedes or follows a speech sound with higher energy (e.g., a vowel; Souza, 2002; Hopkins et al., 2012). At the same time, when fast compression is implemented in a multi-channel HA that processes each frequency channel independently, it tends to reduce spectral contrasts (i.e., by flattening the speech spectrum) and may thus have a deleterious effect on the perception of speech formants, which are crucial for the identification of vowels (Bor et al., 2008). By causing rapid variations of the signal amplitude at the onset and offset of speech sounds, fast compression speeds can also distort the signal envelope and therefore negatively impact speech intelligibility (Stone and Moore, 1992, 2008; Stone et al., 2009). These distortions are more likely to happen when high compression ratios are used (Verschuure et al., 1996). Despite their impact on speech intelligibility, there is currently no consensus as to the best time constants that should be used: time constants used clinically and commercially in hearing aids vary broadly, with attack and release times ranging from 0.5 to 2,000 ms and 10 to 5,000 ms, respectively (Moore and Sȩk, 2016).

The present study extends the work of Gonçalves Braz et al. (2022) by investigating whether OPRA-RS can also be used to optimize time constants. Attack and release times were optimized for HA configurations that corresponded to the compression thresholds and/or the insertion gains recommended either by OPRA-RS or by CAM2 for the same 12 audiometric profiles as used in Gonçalves Braz et al. (2022). As these HA configurations sometimes involved high compression ratios (>3), and that in such cases, fast compression can distort the signal envelope and thus affect speech intelligibility (Souza, 2002), only “slow” compression speeds were used. To assess ASR performance, speech stimuli were first amplified using an HA simulator and then degraded to mimic the perceptual consequences of the elevation of hearing thresholds and loudness recruitment. The resulting speech signals were eventually fed to an ASR system for recognition. The optimization of compression speed was carried out twice in order to assess the reproducibility of the outcomes in terms of ASR scores and optimized time constants.

## Methods

### Overview of the Optimization Chain

**FIGURE 1:**
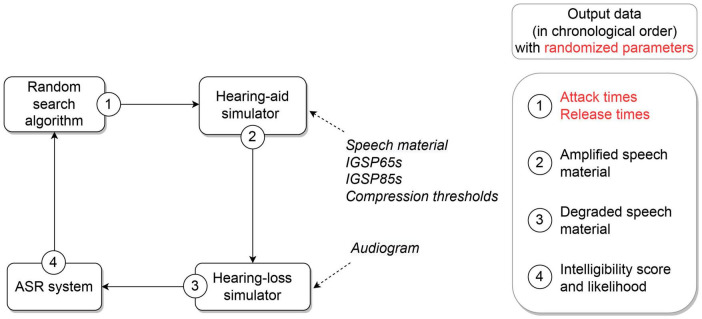
Components of the OPRA-RS optimization chain, with associated input data (in italics) and output data (right panel). The parameters randomized by the RS algorithm are highlighted in red.

Figure 1 describes the processing chain used to optimize time constants for a given input audiogram. At initialization, the RS algorithm randomly selects attack and release times within two ranges of possible values. These time constants, as well as the compression thresholds and the insertion gains prescribed by OPRA-RS or CAM2 for a 65- and 85-dB-SPL speech input level (IGSP65s and IGSP85s, respectively; see Supplementary Datasheet for more details), are transmitted to an HA simulator. The HA simulator amplifies 50 speech stimuli corresponding to five 10-word lists of the speech intelligibility test of Fournier (1951), which is the test most often used by French audiologists for speech audiometry (Rembaud et al., 2017). The amplified speech signals are then degraded by the HL simulator, according to the input audiogram. The resulting speech signals are finally processed by an ASR system developed for the French language.

A total of *N* iterations are used to assess *N* time-constant configurations. After each iteration, the ASR score and average log-likelihood of recognized words yielded by the current time constants are compared to those obtained with the best time-constant configuration found up to the current iteration. If the current configuration yields a higher ASR score (or the same ASR score but with a higher log-likelihood) than the previous best configuration, the current configuration is used as a baseline for the next iteration. Otherwise, the best previous configuration serves as a baseline for the next iteration.

Based on the current number of iterations *i*, the search ranges are reduced around the baseline time constants for the next iteration, following the equation:


(1) $$s\,e\,a\,r\,c\,h\,r\,a\,n\,g\,e\,\left(i+1\right)=s\,e\,a\,r\,c\,h\,r\,a\,n\,g\,e\,\left(i\right)-\frac{i\,n\,i\,t\,i\,a\,l\,s\,e\,a\,r\,c\,h\,r\,a\,n\,g\,e-\left(2\times s\,t\,e\,p\,s\,i\,z\,e\right)}{N}$$

where *stepsize* corresponds to the step (in ms) used to define possible values within the search range.

### Simulation of Hearing-Aid Processing

A 5-channel HA simulator implemented in MATLAB™ (Moore et al., 2010a) was used to amplify the speech signals. The frequency ranges of the five HA channels were 0.1–0.7, 0.7–1.4, 1.4–2.8, 2.8–5.6, and 5.6–8 kHz. In each channel, the simulator used two dynamic range compressors placed in series: the wide dynamic range compression function was applied in the first compressor, while the second compressor was used as a limiter. For further details about the implementation of the HA simulator, see Fontan et al. (2020c).

### Simulation of Hearing Loss

The functioning of the HL simulator, also implemented in MATLAB™, is detailed in Nejime and Moore (1997). As done in Gonçalves Braz et al. (2022), the simulator was used to mimic two of the perceptual consequences of age-related HL: Based on the input audiogram, a linear filter simulated the elevation of hearing thresholds, while loudness recruitment was simulated by raising the signal envelope (Moore and Glasberg, 1993).

### Automatic Speech Recognition System

The ASR system used in the study consisted of Hidden Markov Models and Gaussian Mixture Models. It was implemented using the Julius ASR engine (Lee and Kawahara, 2009). The acoustic models were trained on approximately 100 h of French radio broadcast news. These speech recordings were not processed to mimic HA amplification or HL and did not include the 50 word recordings used in the study to evaluate time constants. The lexicon used by the ASR system only comprised the 50 target words. A more detailed description of the ASR system is given in Gonçalves Braz et al. (2022).

### Test Procedure

**FIGURE 2:**
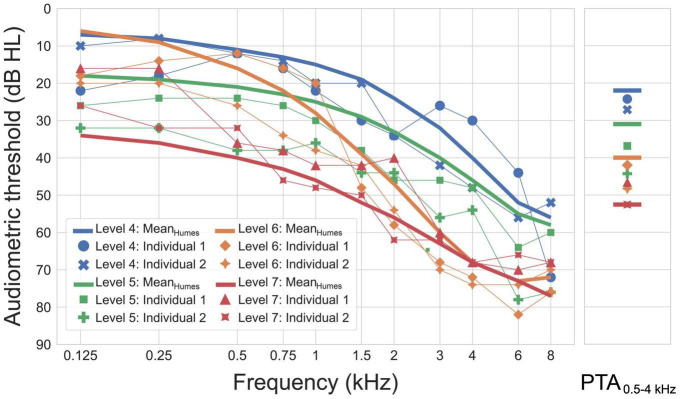
Audiograms used as an input for the simulation of hearing loss. Corresponding pure-tone averages (PTAs) for frequencies between 0.5 and 4 kHz are shown in the right panel. Figure reproduced from Gonçalves Braz et al. (2022).

The processing chain was used to optimize time constants for 12 audiograms, using the compression thresholds selected by OPRA-RS and the insertion gains prescribed either by OPRA-RS or by CAM2. The audiograms, shown in Figure 2, represented mean or individual audiometric thresholds falling into levels 4–7 of the Wisconsin Age-Related Hearing Impairment Classification Scale (WARHICS; Cruickshanks et al., 2020). The audiograms corresponded to mild-to-moderately severe losses, with thresholds generally increasing as a function of frequency, as is typical of age-related HL. The mean audiograms were based on the data collected by Humes (2021). Some of the hearing thresholds required by the HL simulator (corresponding to the frequencies 0.125, 0.25, 0.75, and 1.5 kHz) were not included in the mean audiograms reported by Humes (2021). Those missing thresholds were intra- or extrapolated using third-least-squares polynomial regressions. The individual audiograms corresponded to older patients (mean age: 70 years; age range: 63–78 years) with sensorineural HL. For each of the four WARHICS levels, one mean audiogram and two individual audiograms were used.

The RS algorithm that yielded the highest ASR performance in Gonçalves Braz et al. (2022) was used in the present study. This algorithm tunes all parameters (here, time constants) in all HA channels simultaneously. As in Gonçalves Braz et al. (2022), four independent RS threads were run in parallel. Each thread consisted of 1,000 iterations, during which time constants were randomly varied within predefined search ranges, using 10-ms steps. At the start of the RS, the search ranges were 10–500 ms for attack times, and 300–2,000 ms for release times. These ranges correspond to those generally associated with a slow compression system (Moore, 2008a,b; Moore et al., 2010a; Moore and Sȩk, 2013). For each audiogram, the final time-constant configuration yielding the highest ASR performance across the four search threads was selected. In what follows, unless explicitly mentioned, only data from the first repetition of the RS algorithm are used.

## Results

**FIGURE 3:**
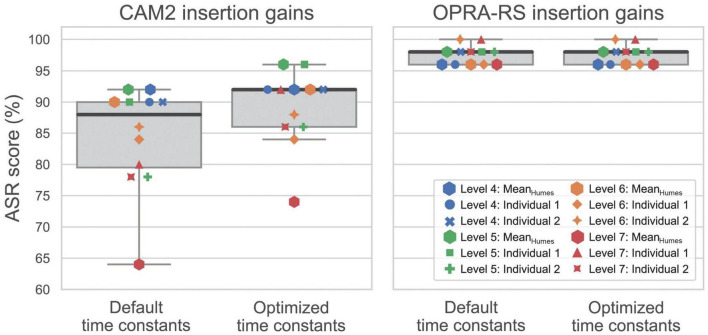
ASR scores with or without optimization of time constants, using the insertion gains recommended by CAM2 **(left panel)** or OPRA-RS **(right panel)** for the 12 audiograms. Horizontal, thick dark lines inside the boxes represent median values. The 0 and 100th percentiles are represented by the bottom and top whiskers, while the bottom and top limits of the boxes represent the 25th and 75th percentiles.

Figure 3 compares the ASR scores achieved with default and optimized time constants, using either the insertion gains recommended by CAM2 (left panel) or those calculated by OPRA-RS (right panel). The default time constants correspond to the fixed compression speeds used by Fontan et al. (2020c) and Gonçalves Braz et al. (2022). Those were 200, 100, 100, 100, and 100 ms for attack times, and 2,000, 1,500, 1,200, 1,000, and 1,000 ms for release times for HA channels 1–5, respectively.

With the insertion gains recommended by CAM2, it can be noticed that ASR scores tended to be higher after the optimization of time constants (median ASR score: 92%) than with default time constants (median ASR score: 88%). As Kolmogorov-Smirnov tests indicated that the ASR scores were not normally distributed (*p* ≤ 0.044 in both conditions), a Wilcoxon signed-rank test was used to assess the significance of the observed difference. The results show that ASR scores are significantly higher after the optimization of time constants (*Z* = 2.8; *p* = 0.005). In contrast with this general trend, for two out of the 12 audiograms, all time-constant configurations tested during the RS yielded lower ASR scores than those obtained with the default constants. The improvements due to the optimization of time constants seem to be larger for the most severe HLs than for milder HLs. For example, for audiograms corresponding to level 7 of the WARHICS scale, the ASR score improved by 10 percentage points on average, whereas an average improvement of 1.3 percentage point is observed for audiograms corresponding to level 4 of the WARHICS scale. A Spearman correlation was computed to assess the existence of a significant association between HL severity, represented by the pure-tone average (PTA) for frequencies of 0.5, 0.75, 1, 1.5, 2, 3, and 4 kHz, and the improvement in terms of ASR score due to the optimization of time constants. The results indicate a significant positive relationship between the two variables (ρ = 0.62; *p* = 0.03), that is, the higher the PTA, the larger the benefit due to the optimization of time constants.

Contrary to the ASR scores obtained with CAM2 gains, no improvement was observed after the optimization of time constants when using the gains recommended by OPRA-RS. For six out of the 12 audiograms, all time-constant configurations tested during the RS yielded lower ASR scores than those obtained with default time constants.

The reproducibility of the ASR scores was assessed by comparing the outcomes of the two repetitions of the RS algorithm. For CAM2, the median ASR score achieved during the second repetition of the algorithm (91%) was very close to the score achieved during the first repetition (92%); a Wilcoxon test revealed that no significant difference existed between the ASR scores yielded by each of the repetitions (*Z* = −1.7; *p* = 0.10). For OPRA-RS, all ASR scores remained equal across repetitions of the RS algorithm.

**FIGURE 4:**
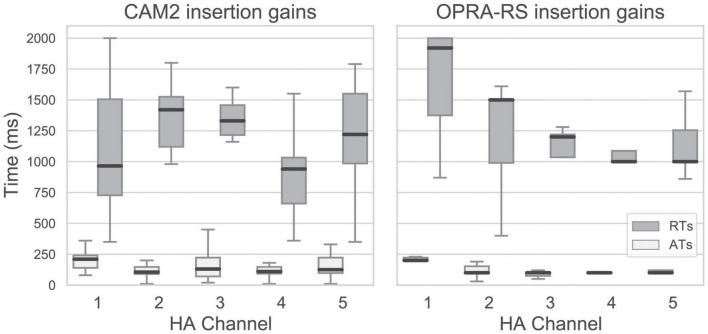
Distribution of the attack times (ATs) and release times (RTs) yielding the highest ASR performances when using the insertion gains recommended by CAM2 **(left panel)** or OPRA-RS **(right panel)** for the 12 audiograms. Otherwise as Figure 3.

Figure 4 shows the distribution of attack and release times yielding the highest ASR performances for the 12 audiograms with the insertion gains recommended by CAM2 (left panel) or by OPRA-RS (right panel). In the cases for which better ASR scores were achieved with the default time constants used by Gonçalves Braz et al. (2022), these default values were retained as best configurations.

Median attack and release times across channels are 135 and 1,190 ms, respectively, for CAM2 gains, and 100 and 1,200 ms, respectively, for OPRA-RS gains. Contrary to attack times, optimized release times span the entire possible range of values. Optimized time constants seem less variable for OPRA-RS than for CAM2. This is at least partially due to the fact that, for OPRA-RS, a larger proportion of the optimized time constants correspond to the default constants used by Gonçalves Braz et al. (2022).

Finally, the time constants obtained during the two repetitions of the RS algorithm were compared. As default time constants corresponded to fixed values, the HA configurations for which the best ASR scores were achieved with default time constants were excluded from this analysis. For the remaining HA configurations (*N* = 16), the median absolute differences across repetitions were 85 and 355 ms for attack and release times, respectively. The minimum and maximum absolute differences were 0 and 420 ms for attack times, and 20 and 1,640 ms for release times.

## Discussion

This study provides proof of concept that RS can be used for the optimization of HA time constants for a given audiometric profile. This approach might prove particularly useful since there is currently no consensus as to the time constants that should be used to maximize speech intelligibility for a HI individual (Moore and Sȩk, 2016). It has been shown that knowledge of the HA user’s cognitive abilities might help to choose slow or fast compression (Gatehouse et al., 2003; Souza and Sirow, 2014), but the results of studies addressing the relationship between hearing abilities and optimal time constants are heterogeneous (Hopkins et al., 2012; Moore and Sȩk, 2016). Within this context, OPRA-RS represents a novel approach that, given the audiometric profile of the HA user, can be used to systematically explore a large number of time-constant configurations and assess their impact in terms of speech intelligibility.

ASR scores for the optimized time constants were reproducible across repetitions of the RS algorithm, but were associated with different combinations of time constants. This is possibly due to an interaction between attack and release times, as the two parameters were optimized simultaneously. Future studies should optimize each parameter independently to assess their reproducibility. It might also be interesting to extend in future studies the search ranges used for attack and release times, which were limited in the present study to values generally associated with slow compression.

For the mild-to-moderately-severe HLs used in the this study, the optimization of time constants yielded significant improvements in ASR scores for CAM2, but not for OPRA-RS. In addition, the improvements observed for CAM2 were small (4 percentage points, corresponding to 2 out of the 50 words used in the study). These observations are likely due to ceiling effects in the two test conditions, even before the optimization of time constants. Indeed, it was observed that more severe HLs, yielding the lowest ASR scores with CAM2 and default time constants, were associated with higher improvements after the optimization of time constants. To limit such ceiling effects and thus to assess if clinically significant benefits can be obtained, future studies should use more challenging experimental conditions (e.g., speech materials that are shorter and/or presented in noise).

Finally, it should be determined if, as shown by Fontan et al. (2020c) for the fine-tuning of insertion gains, the benefits observed in ASR performance due to the fine-tuning of time constants translate into speech-intelligibility benefits for actual listeners with age-related HL, and if these benefits are clinically relevant. Also, in the present study, CAM2 as a baseline prescription since it was used in the previous experiments on ASR-based optimization of HA parameters (Fontan et al., 2020c; Gonçalves Braz et al., 2022). It should be determined if significant improvements are also observed for those prescription rules that are more widely used in clinical practice, such as NAL-NL2 (Keidser et al., 2011).

## Data Availability Statement

The datasets generated and analyzed for this study can be obtained from the corresponding authors for any research purpose.

## Author Contributions

LF initiated the idea. LG designed and implemented the random-search algorithms, under the supervision of JP. MS provided scientific advice about fitting algorithms, and the hearing-aid and hearing-loss simulations. LF, LG, MS, and CF analyzed and interpreted the data. LF and CF wrote the manuscript. All authors approved the final version of the manuscript.

## Conflict of Interest

This study is part of the development of a future product/service by Archean LABS intended for hearing-aid audiologists. CF acted as a scientific consultant. The remaining authors declare that the research was conducted in the absence of any commercial or financial relationships that could be construed as a potential conflict of interest.

## Publisher’s Note

All claims expressed in this article are solely those of the authors and do not necessarily represent those of their affiliated organizations, or those of the publisher, the editors and the reviewers. Any product that may be evaluated in this article, or claim that may be made by its manufacturer, is not guaranteed or endorsed by the publisher.
